# Innovative High-Throughput SAXS Methodologies Based on Photonic Lab-on-a-Chip Sensors: Application to Macromolecular Studies

**DOI:** 10.3390/s17061266

**Published:** 2017-06-02

**Authors:** Isaac Rodríguez-Ruiz, Dimitri Radajewski, Sophie Charton, Nhat Phamvan, Martha Brennich, Petra Pernot, Françoise Bonneté, Sébastien Teychené

**Affiliations:** 1 CEA, DEN, DMRC, SA2I, 30207 Bagnols-sur-Cèze, France; isaac.rodriguez-ruiz@cea.fr (I.R.-R.); sophie.charton@cea.fr (S.C.); 2 Laboratoire de Génie Chimique, UMR 5503, 4 allée Emile Monso, 31432 Toulouse, France; dimitri.radajewski@ensiacet.fr (D.R.); nhatpv.coltech@gmail.com (N.P.); 3 European Molecular Biology Laboratory, 71 avenue des Martyrs, 38000 Grenoble, France; martha.brennich@esrf.fr (M.B.); rejma@esrf.fr (P.P.); 4 Institut des Biomolécules Max-Mousseron, UMR 5247, Université d’Avignon, 33 rue Louis Pasteur, 84000 Avignon, France; francoise.bonnete@univ-avignon.fr

**Keywords:** Photonic Lab-on-a-Chip, spectrophotometric detection, SAXS, microfluidics, protein interactions

## Abstract

The relevance of coupling droplet-based Photonic Lab-on-a-Chip (PhLoC) platforms and Small-Angle X-Ray Scattering (SAXS) technique is here highlighted for the performance of high throughput investigations, related to the study of protein macromolecular interactions. With this configuration, minute amounts of sample are required to obtain reliable statistical data. The PhLoC platforms presented in this work are designed to allow and control an effective mixing of precise amounts of proteins, crystallization reagents and buffer in nanoliter volumes, and the subsequent generation of nanodroplets by means of a two-phase flow. Spectrophotometric sensing permits a fine control on droplet generation frequency and stability as well as on concentration conditions, and finally the droplet flow is synchronized to perform synchrotron radiation SAXS measurements in individual droplets (each one acting as an isolated microreactor) to probe protein interactions. With this configuration, droplet physic-chemical conditions can be reproducibly and finely tuned, and monitored without cross-contamination, allowing for the screening of a substantial number of saturation conditions with a small amount of biological material. The setup was tested and validated using lysozyme as a model of study. By means of SAXS experiments, the proteins gyration radius and structure envelope were calculated as a function of protein concentration. The obtained values were found to be in good agreement with previously reported data, but with a dramatic reduction of sample volume requirements compared to studies reported in the literature.

## 1. Introduction

Small-Angle X-ray scattering (SAXS) is known to provide very useful information to investigate the structure of soft matter and biological macromolecules at the nanometer scale [1,2]. It has been proven to be a powerful technique in a vast number of applications, ranging from nucleation studies [3,4] to macromolecular interactions, leading to the determination of protein molecular weight [5], structural information [6], or protein conformational changes [7]. However, due to the large number of experiments required to obtain reliable statistical information, this technique usually becomes less convenient when working with high value compounds. 

The miniaturization of the experimental setups (and therefore of sample requirements) for SAXS measurements presents obvious advantages, and few recent studies have proposed the use of continuous flow microfluidic platforms to perform studies with macromolecules and other expensive compounds [8,9,10]. Here, the use of droplet-based microfluidic platforms, although sparsely reported at the present time [11,12,13], is clearly advantageous. Droplet-based microfluidics essentially consists in the co-flow of two immiscible liquid phases in a microfluidic channel [14]. This co-flow causes the alternate generation of droplets of the two phases and, in contrast to continuous phase flows (where all reagents and products of reaction are able to diffuse in the channels, thus affecting the whole system), this permits the creation of thousands of independent monodisperse experiments in a short period of time. Each droplet thus acts as an isolated reactor with the advantage of using a very low quantity of reagents. This is particularly convenient, not only for high value products, but also when considering working with hazardous materials.

In the abovementioned context, the possible integration of transducers at the microfluidic scale, yet unexplored, can provide extremely valuable experimental information. Among the different transduction systems (such as magnetic [15], electrochemical [16], or photonics [17]), optical transducers, leading to the Photonic Lab-on-a-Chip (PhLoC) paradigm, have demonstrated to be one of the most sensitive and selective analytical detection methods, while providing the advantageous property of preserving sample properties (such as sterility, of high relevance for biological applications) due to its noncontact measurement principle [18]. In this regard, the non-invasive on-chip characterization of biological and chemical responses by UV-Vis spectrophotometry has led to numerous PhLoC and optofluidic sensors [19,20]. These systems have been proposed for a wide range of applications, from cell culturing and cell analysis [21,22,23,24] to heavy metal ion detection [25,26], enzymatic catalysis for different applications [27,28], and protein concentration measurements [29]. More particularly, this technology can be of special interest to investigate reactions involving radioactive elements, (e.g., for nucleation studies [30], mass transfer [31], etc.). Therein, and thanks to the on-chip sensor integration, the readout can be advantageously remote from the measured zone and be connected to the PhLoC, minimizing any risk related to radiation [32].

In a previous work, we recently demonstrated the convenience of coupling SAXS and high throughput microfluidic techniques to study protein crystallization from solution [11]. In this work, we propose a further improvement on this methodology by taking advantage of photonic sensing implementation by means of Photonic Lab-on-a-Chip technology. Hence, the coupling of (i) high throughput droplet-based microfluidics, (ii) on-chip spectrophotometric detection for real-time protein concentration measurements, and (iii) SAXS, for the realization of molecule and molecular interactions studies, is here proposed by means of a PhLoC platform. To validate both setup and methodology, lysozyme, a well-known protein widely characterized in the literature, is proposed as a model of study. The implementation of photonic detection provides a two-fold function in this setup. First, it allows for the transformation of the biological/chemical information contained in each droplet into a quantifiable concentration signal and, second, also provides complementary and useful data regarding droplet size, stability, and generation frequency, thus facilitating subsequent SAXS measurements synchronization and data interpretation. 

## 2. Materials and Methods

### 2.1. Reagents

Lysozyme was purchased from Sigma-Aldrich (dialyzed and lyophilized 629710). Initial protein solutions were prepared by solving lysozyme in sodium acetate buffer (50 mM sodium acetate, pH 4.4) prepared with Milli-Q water (Millipore, Billerica, MO, USA). Prior to use, the lysozyme solutions were prepared using lysozyme, crystallized and dialyzed four times, either through a preparative column or filtered through Amicon ultra filters, in order to remove any unwanted aggregates present in most of the commercially available lysozyme preparations [33]. Water-in-oil droplets of protein solution at different concentrations (by on-chip dilution of a mother solution using acetate buffer) were generated and transported by an immiscible fluorous oil (Krytox GPL100, DuPont, Wilmington, DE, USA) containing 2% w/w of fluorinated surfactant (tri-block copolymer, PFPE-PEG-PFPE, purchased from RAN Biotechnologies, Beverly, MD, USA) in order to stabilize the droplets interface. The selection of both oil and surfactant was made considering a compromise between good resistance to X-ray radiation damage, an optimal viscosity, and immiscibility with the aqueous phase, as previously reported [11].

### 2.2. PhLoC Design and Fabrication

PhLoC platforms, with rectangular channels of cross section of 280 × 300 µm^2^, were fabricated using standard soft lithography and cast molding techniques. An inexpensive multilevel negative tone photoresist dry film (WBR2000 series, DuPont, Wilmington, DE, USA) was laminated on a glass substrate (Thermo Scientific Menzel-Glaser, Braunschweig, Germany) following a procedure described elsewhere [34]. Summarizing, the designed PhLoC configuration was patterned by UV exposure (UV-KUB2, Kloé, Montpellier, France) through a low cost emulsion mask, and structures were subsequently developed using sodium carbonate (Na_2_CO_3_) 1% and rinsed by an aqueous solution of magnesium sulfate (MgSO_4_) 0.5%. In addition, the dry film structures were treated with toluene to achieve hydrophobic surface properties before PhLoC replicas were obtained by casting PDMS (Sylgard 184 elastomer kit, supplied by Dow Corning, MI, USA) in the dry film master mold. Finally, PDMS surfaces were activated using a corona treater (BD-20AC, Electro-Technic Products Inc., Chicago, IL, USA) and bonded to a glass substrate, thus sealing the microfluidic structures [35].

### 2.3. Experimental Setup and PhLoC Configuration

**Figure 1:**
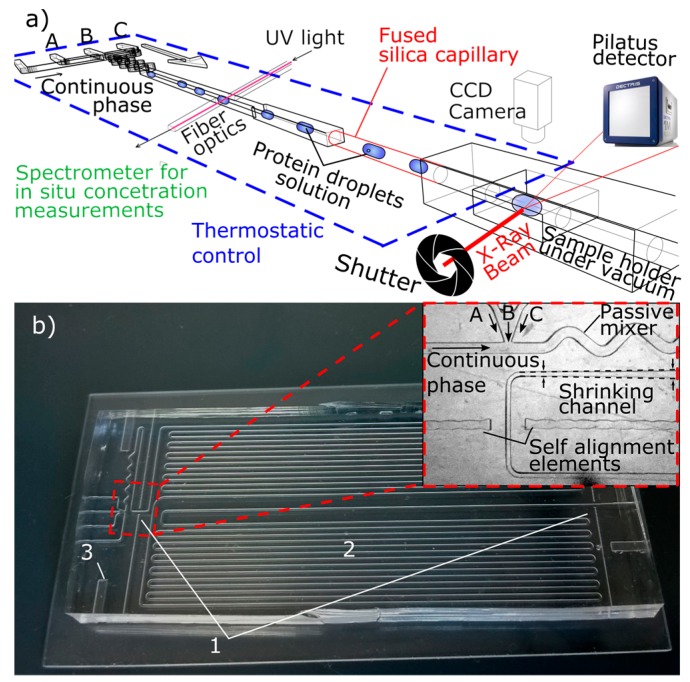
(**a**) Schematics of the PhLoC-SAXS configuration. Protein solution droplets at different concentrations are generated and monitored by continuous sensing in the PhLoC platform. Subsequently, they are sent to the SAXS sample holder, where measurements are synchronized with the droplets in movement by actuating in the beam shutter. (**b**) Picture and details of the PhLoC platform showing (1) interrogation areas for photonic detection, comprising two self-alignment elements for pig-tailed fiber optics positioning, (2) serpentine channel for droplet storage, and (3) inlets for temperature probes. Inset show details of the mixing and droplet generation area, comprising 3 channels for reagent injection (A, B, C), an extra channel for continuous phase injection, and a passive zigzag mixer allowing effective and fast droplet homogenization.

**Figure 2:**
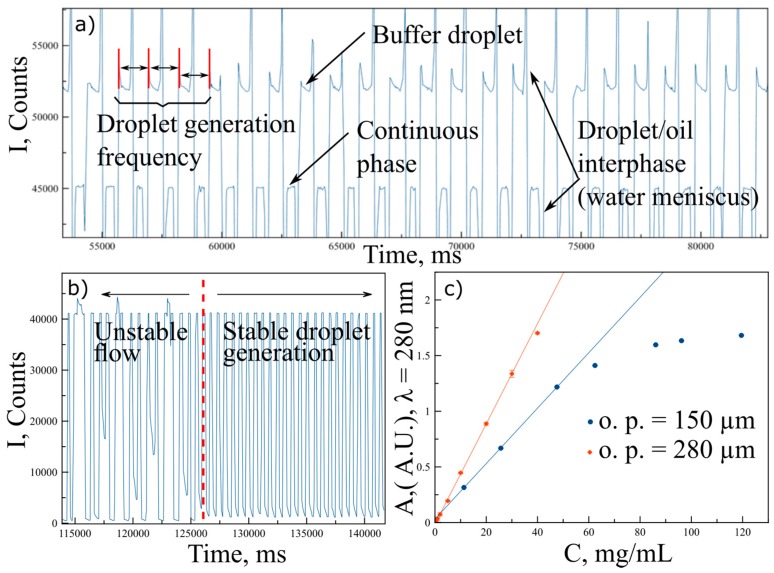
(**a**) Typical intensity spectra collected at *λ* = 280 nm, when operating with the PhLoC generating stable droplets, at constant flow rates. The continuous phase is observed at a constant intensity value while the dispersed aqueous phase can present different intensity values as a function of protein absorbance at the given wavelength. (**b**) Intensity spectrum resulting from monitoring the first instants of droplet generation. Red dashed line separates the spectrum into two regions: Left part shows the spectra of different unsteady droplets, leading to different intensity signals at *λ* = 280 nm, in accordance to their protein concentration. Right part shows steady droplet generation with homogeneous and reproducible protein concentration. (**c**) Absorbance calibration plots at *λ* = 280 nm for lysozyme measured on chip through a 150 µm (red diamonds) and a 280 µm (blue circles) optical paths. Lines depict the concentration linear range for each plot.

Figure 1a depicts a schematic of the experimental setup. The operation of the PhLoC platform envisages the injection of up to 3 different aqueous solutions (A, B, and C in Figure 1a,b) together with an inert and immiscible continuous phase to generate droplets. Reagent solutions (protein solution and buffer) were injected into the PhLoC at controlled flow rates by means of high precision syringe pumps (neMESYS Cetoni, Korbußen, Germany) coupled to 1 mL syringes (Hamilton, Reno, NV, USA). Different flow rates ratios provide droplets with different protein concentrations. After generation, droplets are quickly homogenized by means of a passive zigzag mixer, and spectrophotometrically monitored (Figure 1b-1) before and after droplets storage for tempering into the PhLoC serpentine channel (Figure 1b-2). This detection configuration allows for monitoring of any possible phase transition (in case of crystallization if supersaturated solutions are analyzed) and for the droplet stability to be checked after the serpentine. For this purpose, optical interrogation areas were located perpendicularly to the microfluidic channel. The coupling of light to the microfluidic structure was achieved by means of pig-tailed 220 µm solarization-resistant fiber optics (Thorlabs, Newton, NJ, USA, NA = 0.22). Self-alignment elements were designed for fiber optics accurate positioning enabling an optimal light coupling–decoupling to the system. A 5 W halogen AvaLight-D(H)-S light source and an Avaspec 2048-USB2 spectrometer (Avantes, Apeldoorn, Netherlands) were used for light coupling and subsequent spectrum analysis, and absorbance measurements were performed at *λ* = 280 nm to determine protein concentration [29]. In order to obtain a spectrophotometric time-resolution allowing for a correct monitoring of droplets flowing through the PhLoC microfluidic channels, spectra were collected at the shortest possible integration times. Hence, to ensure an adequate signal-to-noise ratio, micro-lenses for light beam collimation were excluded for this detection configuration, thus enhancing light coupling efficiency: considering the short optical paths for analyte interrogation and the small numerical aperture of the fiber optics, the light losses caused by Fresnel reflections in any microlens are found to be more important than the dispersion of light due to beam divergence [34].

**Figure 3:**
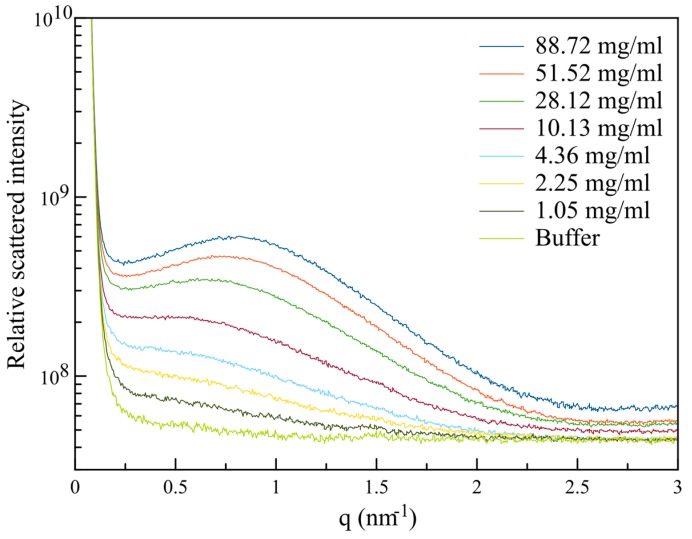
Normalized scattered intensity of lysozyme in NaAC 50 mM buffer pH 4.5 at different concentrations.

For a precise control of the molecule saturation state in the droplets, the PhLoC platform was held in a Peltier-based thermostated plate. By thermostatizing the serpentine structure, it is possible to tune droplets reaction times (induction times for crystallization in case of supersaturated solutions) after quenching, before their analysis. The PhLoC integrates lateral openings for temperature probes insertion (Figure 1b-3) controlling the temperature in the thermostated plate within the range from 0 to 50 °C.

Links between the PhLoC platform and the SAXS sample holder were made by connecting a flexible fused silica capillary (ID 280 µm, OD 360 µm, Postnova Analytics, Landsberg am Lech, Germany, depicted in red in Figure 1a) directly to the exit of the microfluidic platform and to the quartz capillary (OD 300 µm, wall thickness 10 µm) of the sample holder. The latter was hermetically sealed in order to keep vacuum at a residual pressure ~10^−2^ mBar for obtaining high-quality SAXS data. Droplets stored in the PhLoC serpentine channel were sent to the SAXS detection area at a desired speed (and through the fused silica capillary), by injecting continuous phase into the PhLoC platform at a controlled flow rate. Temperature was also maintained constant along the connecting capillary by means of a thermostated bath and external tubing surrounding the capillary. 

### 2.4. SAXS Experiments

Synchrotron SAXS measurements were performed on the beamline BM29 at the European Synchrotron Radiation Facility (ESRF) in Grenoble, France [36]. A 1 M Pilatus detector was used to record the two-dimensional SAXS patterns at an experimental X-ray wavelength of 0.0991 nm and a distance sample-to-detector of 2.87 m. In this configuration, the scattering vector q = 4πsinθ/λ covered a range of 0.03–4.5 nm^−1^. A 90 µm (vertically) × 165 µm (horizontally) beam cross section, ensuring an interrogation area smaller than the droplets generated by the PhLoC platform, was defined by slits at the sample plane. The sample holder can be translated with respect to the X-ray beam by a few millimeters with a precision of ten microns, thus allowing a perfect alignment of the capillary. In order to minimize radiation damage and temperature changes in the protein solution, and collecting only SAXS measurements from the center of each droplet, the X-ray beam shutter was synchronized with the droplets, flowing through the quartz capillary, by means of a CCD camera and real time image processing, using an ad-hoc MATLAB application connected to a TLL pulse generator. Due to the small beam size, the exposure time used in the experiments (100 ms for each SAXS acquisition), and the nature of the aqueous sample, temperature fluctuations during measurements, due to the highly energetic nature of the beam, could be considered negligible in our experimental setup [37].

## 3. Results and Discussion

### 3.1. On Chip Real-Time Spectrophotometric Detection

Figure 2a shows the typical intensity spectrum collected when a solution containing NaAc 50 mM buffer pH 4.5 (reference blank for lysozyme absorbance measurements measured at *λ* = 280 nm) was injected generating monodisperse droplets with the continuous immiscible phase. In this spectrum, we can observe both continuous and aqueous phase, giving a very constant intensity values (measured at the center of the droplets), and maximum and minimum light intensity peaks due to light beam interaction with the meniscus corresponding to the aqueous/oil interface. Additionally, it is also possible to observe the droplets as a function of time, obtaining a good measurement of droplet size (once flow rates are determined and fixed) and droplet generation frequency. Moreover, the stability of this pattern described by the intensity spectra allows to discriminate when droplets generation is unstable (mainly when flow rates are not stabilized) or when droplets are steadily generated (Figure 2b), thus leading to homogenous droplets, which population number and concentration can be straightforwardly determined. According to the protein concentration range to be measured, it becomes necessary to consider a minimum optical path ensuring enough sensitivity. However, additionally, in accordance to the Beer–Lambert law, when decreasing the optical path, absorbance signal decreases as well, so it is also possible to extend the absorbance linear range at higher concentrations [29]. Hence, for the work here presented, in which protein interactions for lysozyme at mid to high concentrations are explored, PhLoCs with two different optical paths for droplet interrogation have been considered. The first one was designed with a constant channel width of 280 µm, while the second one was provided with a gradually shrinking channel to reach an interrogation optical path of 150 µm (inset in Figure 1b). Figure 2c shows the corresponding calibration plots for lysozyme. Each absorbance point in this plot represents an average of 20 droplets, revealing in each case a very high reproducibility, with a standard deviation of ~0.001 absorbance units. As can be observed, a decrease in the optical paths provides a decrease in the absorbance signal for the same measured concentrations, with a consequent extension in the linear range.

It is worth emphasizing that this methodology, based on light extinction measurements (absorbance in this case), can be applied not only for measuring protein concentration at the UV, but also for droplet monitoring of any system displaying absorption and/or scattering properties in the UV-Vis spectrum. Both versatility and sensitivity of the technique are straightforwardly tunable by selecting the most convenient optical paths and wavelength(s) to monitor a given analyte [29].

### 3.2. Macromolecular Interactions Characterization by SAXS

Small angle X-ray scattering has become a particularly convenient technique for preliminary studies regarding protein shape and conformation [38]. It is also convenient to study the interactions between proteins and other molecules in solution, either below saturation state or newly supersaturated, though not yet crystallized [39]. In order to validate the present experimental methodology, SAXS data obtained at different protein concentrations was analyzed to obtain the protein radius of gyration, *R_g_* which is a numerical indicator of the protein structure compactness. *R_g_* value has been previously reported for lysozyme (1.43 ± 0.04 nm) [34]. The latter was determined using sample volumes up to three orders of magnitude higher than the ones required involving microfluidic tools, as experimented here.

When considering monodisperse spherical molecules/particles, the total intensity *I(c,*$\vec{q}$*)* scattered at a scattering angle 2*θ*, can be expressed as a function of the scattering vector $\vec{q}$ (where $\vec{q}$ = 2sin *θ/λ*), by the following equation:

(1) $$\;\;I\left(c,\;\vec{q}\right)=I\left(0,\;\vec{q}\right)\cdot \;S\left(c,\;\vec{q}\right)$$

where the intensity scattered in the absence of interactions is represented by the form factor *I*(0,$\vec{q}$), and the interactions are characterized by the solution structure factor, *S*(c,$\vec{q}$) [40]. Within a smaller $\vec{q}$ range, Equation (1) can also be considered valid for quasi-spherical and/or polydisperse particles. Here, by theoretical simulation of the structure factors, we can determine the best-fit parameters of molecule/particle (protein) interaction potentials [30] and reconstruct the corresponding protein molecular envelope, which gives an estimation of its overall fold and atomic structure. Figure 3 shows the intensity curves, *I(c*,$\vec{q}$*)*, measured, as a function of the scattering vector. Each of these curves was obtained considering an average of at least 100 individual nano-droplets, of reproducible volumes, and exhibiting exactly the same lysozyme concentrations, as evidenced and guaranteed thanks to the on-chip spectrophotometry monitoring preformed upstream of the SAXS beam. Subtracting the (background) buffer signal, and scaling on the same relative value (by normalizing the intensity curves as a function of their corresponding protein concentration, *c*, and direct beam intensity [40]), the previously mentioned form factor of the protein can be determined using the intensity curves recorded at the lowest concentrations.

Here, the protein radius of gyration, *R_g_* can be related to the form factor and the scattering intensity at zero angle, $I_{0}$, by means of the Guinier’s law [41]:

(2) $$I\left(0,\;\vec{q}\right)≅I_{0}exp\left(-\frac{{\vec{q}}^{2}R_{g}^{2}}{3}\right)$$

**Figure 4:**
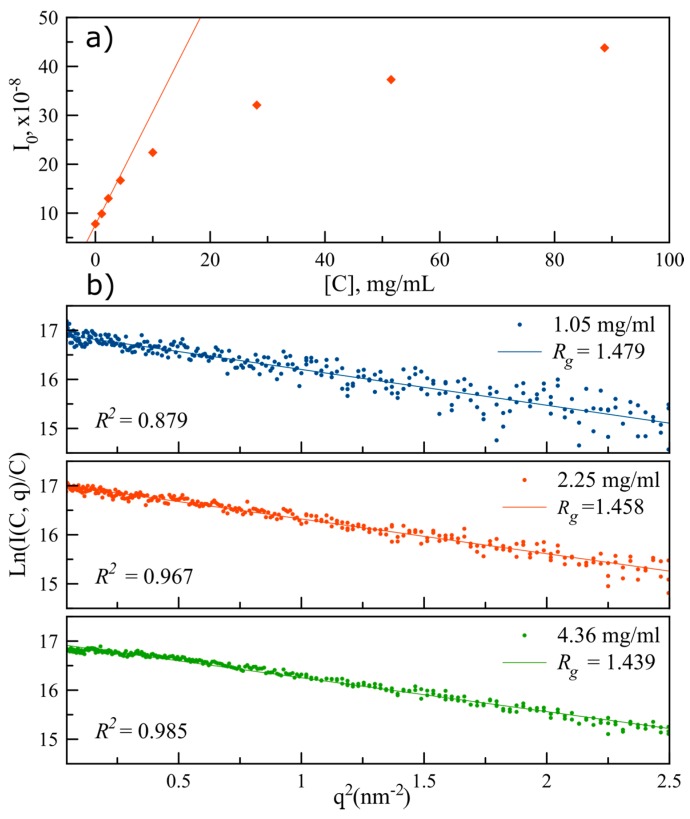
(**a**) Scattered intensity at zero angle as a function of lysozyme concentration. Line represents the region in which the intensity is proportional to protein concentration. (**b**) Least squares fitting of the Guinier plot; *R_g_* and *R^2^* values calculated using three different protein concentrations in the scattered intensity linear region.

Thus, the *R_g_* value for lysozyme can be derived by plotting Ln ($I\left(0,\;\vec{q}\right))$ as a function of the square of the scattering vector (Guinier plot). Additionally, by representing $I_{0}$ as a function of the protein concentration (Figure 4a), a range of concentrations in which a linear relation prevails is observed, where the protein’s scattering intensity is proportional to the concentration, meaning that molecules do not interact with each other (in this particular concentration ranges and given temperature conditions, e.g., 20 °C). Hence, in this linear region, the higher the concentration, the higher the signal-to-noise ratio of the background-subtracted data. When moving to higher concentrations, the distance between individual molecules diminishes and becomes of the same order of magnitude as the intramolecular distances, thus contributing to the scattering pattern (multibody interactions), which results in a loss of linearity. Hence, to test the sensitivity of the technique, the lysozyme form factor was calculated considering the experiences performed in the linear intensity range, which is close to the limit of detection of the PhLoC optical paths configurations [29]. Subsequently, *R_g_* was determined for each form factor by a least squares fitting of the Guinier plot.

*R_g_* values are presented in Figure 4b, together with the corresponding least squares coefficient of determination, *R^2^*, with a view to quantify data dispersion in each experience. As expected, the dispersion of data increases as we approach to the minimal measured protein concentration (1.05 mg/mL) due to the decrease of the signal-to-noise ratio in the data. Nevertheless, all the calculated *R_g_* values exhibit very good agreement with the data previously reported in the literature [42], therefore validating our high-throughput droplet-based PhLoC methodology.

**Figure 5:**
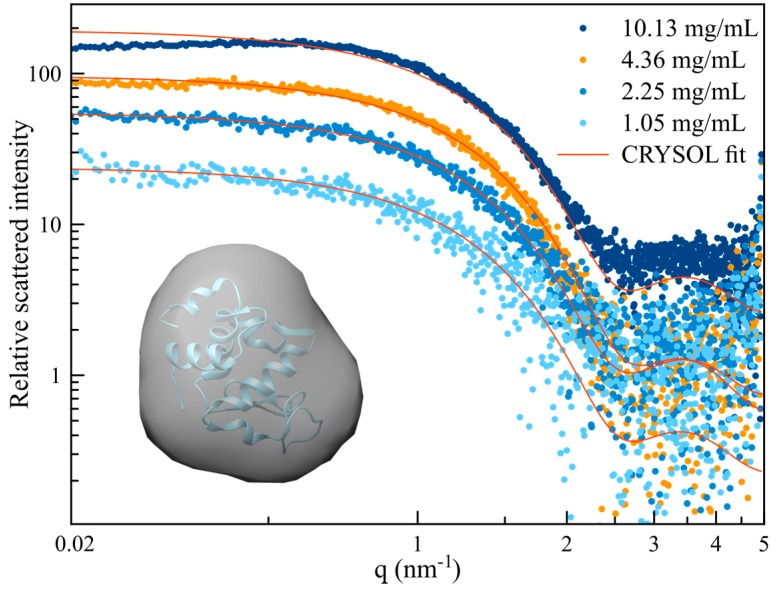
Experimental and calculated scattering intensity using CRYSOL for different protein concentrations. Inset shows the calculated low resolution molecular structure of lysozyme (*χ*^2^ = 0.75), obtained from the scattering data for a concentration of 4.36 mg/mL, depicted in orange in the graph.

Beyond the experimental methodology validation, it remains to be demonstrated that the data acquired using minute amounts of protein are convenient to calculate the protein’s low resolution ab initio structures (i.e., the molecular envelope). Indeed, molecular envelopes can give valuable information regarding protein folding conformation, as well as for crystallographic structure resolution [6,43]. For this purpose, the scattered intensity we obtained for concentrations ranging from 1.05 to 10.13 mg/mL were simulated using CRYSOL software [44] from the atomic coordinate of lysozyme (obtained from the Protein Data Bank structure file 1dpx.pdb). The resulting curves are compared to the experimental ones in Figure 5. A poor agreement is observed for the lowest and the higher concentrations. For the lowest concentration, the signal-to-noise ratio is too low to provide a good fit (*χ^2^* = 2.87). For the highest concentration, in the lower q region (i.e., *q* < 0.5 nm^−1^), the form factor of the lysozyme is higher than the obtained scattering intensity. This means that, at this concentration, the lysozyme solution cannot be considered a diluted solution and that the obtained scattering intensity results from multibody interactions, as observed in Figure 4a. As the experimentally obtained scattering intensity is lower than the form factor, the interactions between lysozyme are repulsive.

The best *χ^2^* value (closest to 1) was obtained for the concentration of 4.36 mg/mL (*χ^2^* = 0.91). For this concentration, the scattering intensity was used to calculate the low resolution ab initio structure (i.e., the molecular envelope) using DAMMIF [45] and DAMAVER [46] software embedded in ATSAS program. The resulted envelope, looking consistent with the actual structure of lysozyme, is presented, together with the protein structure, in the inset of Figure 5.

## 4. Conclusions

The combination of Small-Angle X-Ray Scattering (SAXS), spectrophotometric detection techniques and high throughput, droplet-based microfluidics, is here demonstrated as a powerful tool to investigate molecular and particle interactions. A PhLoC platform was designed for droplet generation, allowing for the monitoring of protein concentration in each monodisperse droplet as well as droplet generation frequency. The droplet flow was synchronized to perform synchrotron radiation SAXS measurements in individual droplets, each one acting as an isolated microreactor, to probe protein interactions while minimizing radiation damage. Additionally, the whole setup was thermostated in order to reach a fine control on molecule saturation state in solution. The setup performances were demonstrated using lysozyme as a model of study, and the sensitivity of the system was tested by determining protein gyration radius and molecular envelope, calculated as a function of protein concentration (obtained by on-chip on-line spectrophotometric measurements) and SAXS experiments. Obtained values were found to be in good agreement with data previously reported in the literature, thus validating the proposed methodology, which provides a dramatic reduction of sample volume requirements and therefore of biological material (or, potentially, other compounds of interest).

Based on these results, and due to the evident advantages provided by volume reduction in terms of sample consumption and high throughput, the methodology appears promising for the study of other systems involving high value or radioactive materials.
